# Supplementary material for: The Impact of a Nutritional Intervention Program on Eating Behaviors in Italian Athletes
Source: Int J Environ Res Public Health. 2021 Jul 8;18(14):7313. doi: 10.3390/ijerph18147313 (PMC8303861; doi:10.3390/ijerph18147313)

PART I

-Did you take part at the first meeting? □ YES □ NO

- Did you take part at the second meeting? □ YES □ NO

- Date of birth __ / __ / ____ - Sex □M □F

- Profession _____________________________________

- Athletic discipline (indicate all disciplines)

____

- Which of these do you use more?

□ by foot	□ by bike	□ by motorcycle	□ by car	□ by bus/train/metro	□ Other _____________

- School/Work/University timetable *(ex. Monday: school from 8:30 to 13:00 ...):*

monday____

thuesday____

wednesday____

thrusday____

friday____

saturday____

sunday____

- Training timetable *(ex. Monday from 16:30 to 18:00, Tuesday rest, Sunday often races / racing from 10:00 to 13:00...):*

monday____

thuesday____

wednesday____

thruesday____

friday ____

saturday____

sunday____

- Have you ever been visited by a nutritionist/dietician before? □ YES □ NO

- Are you currently monitored by nutritionist/dietician? □ YES □ NO

If not, have you ever been monitored by nutritionist/dietician? □ YES □ NO

- Are you allergic/intolerant to something? □ YES □ NO

If yes, which allergy or intolerance? ____

- Do you take drugs/medications regularly? □ YES □ NO

If yes, which one? ____

- Are you used to take supplements (vitamin, minerals, enzymes..)? □ YES □ NO

If yes, which one? ____

- Are you used to drink energy drinks? □ YES □ NO

If yes, which one? ____

- Where do you usually eat your meals? (Do not consider special occasion)

	HOME	CANTEEN/OFFICE	CAFETERIA/BAR/FAST FOOD	RESTAURANT/PIZZERIA	OTHER (Example)
BREAKFAST	□	□	□	□	□_________
SNACK	□	□	□	□	□_________
LUNCH	□	□	□	□	□_________
SNACK	□	□	□	□	□_________
DINNER	□	□	□	□	□_________
OTHER	□	□	□	□	□_________

- When you train, you used to eat (indicate how much before/after):

□ Before ____

□ After ____

□ Before and after ____

□ Other____

- When you race, you used to eat (INDICATE how much before/after):

□ Before ____

□ After ____

□ Before and after ____

□ Other ____

- Food choice:

□ Omnivorous (all food types)	□ Vegetarian	□Vegan	□ Other *(ex. macrobiotic, ethic/religious choices...)* ____

PART II: EATING HABITS

You should complete this part in relation to your HABITS and NOT in relation to special events.

1) Do you have breakfast?

□ Daily	□ 3-4 per week	□ 1-2 per week	□ Never

a) If YES , what do you eat? ____

b) If NOT, why?

□ I'm not hungry	□ I'd like it, but I have no time	□ Other ________________

2) Do you have snack in the morning?

□ Daily	□ 3-4 per week	□ 1-2 per week	□ Never

a) If YES, what do you eat? ____

b) If NOT, why?

□ I'm not hungry	□ I'd like it, but I have no time	□ Other _________________

3) What do you eat for lunch? (More than one choice is possible)

□ Both first and second dishes	□ Only first dish	□ Only second dish	□ Vegetables	□ Bread	□ Fruit	□ Dessert	□ Coffee	□ Other ___________

4) Do you have snacks in the afternoon?

□ Daily	□ 3-4 per week	□ 1-2 per week	□ Never

a) If YES , what do you eat? ____

b) If NOT, why?

□ I'm not hungry	□ I'd like it, but I have no time	□ Other _________________

5) What do you eat for dinner? (More than one choice is possible)

□ Both first and second dishes	□ Only first dish	□ Only second dish	□ Vegetables	□ Bread	□ Fruit	□ Dessert	□ Coffee	□ Other ___________

6) Do you eat more than 5 times daily (breakfast, lunch, dinner, snacks)?

□ Daily	□ 3-4 per week	□ 1-2 per week	□ Never

7) How often do you eat grains (pasta, rice, gnocchi, soup, barley, spelt..)?

□ 2 daily (lunch and dinner)	□ 1 daily	□ 3-4 per week	□1-2 per week	□ Never

8) Which of these food do you eat? (More than one choice is possible)

□ Bread	□ Cracker	□ Rice cakes	□ Toast	□ Other _______________

a) How often daily (for example 1 bread, 1 pocket)?

□ 1 daily	□ 2-3 daily	□ 3-4 daily	□ > 4	□ Occasionally	□ Never

9) How often do you eat meat (chicken, turkey, rabbit, beef, pork…)?

□ 2 daily (lunch and dinner)	□ 1 daily	□ 3- 4 per week	□ 1-2 per week	□ Never

10) How often do you eat cold cuts (ham, bresaola, salami, ecc)?

□ 2 daily (lunch and dinner)	□ 1 daily	□ 3- 4 per week	□ 1-2 per week	□ Never

11) How often do you eat fish (for example cod, salmon, sole, sea bass, shrimps...)?

◻ 2 daily (lunch and dinner)	◻ 1 daily	◻ 3- 4 per week	◻ 1-2 per week	◻ Never

12) How often do you eat cheese (es. parmesan, mozzarella cheese, ricotta cheese, crescenza, taleggio ecc)?

◻ 2 daily (lunch and dinner)	◻ 1 daily	◻ 3- 4 per week	◻ 1-2 per week	◻ Never

13) Do you use parmesan? ◻ YES ◻ NO
a) If YES, how many spoons? (1 teaspoon= ½ spoon) daily?

◻ 1 teaspoon	◻ 2 teaspoons	◻ 3 teaspoons	◻ > 3 teaspoons

14) How often do you eat eggs (es. hard-boiled eggs, omelette, scrambled eggs...)?

◻ 1-2 eggs per week	◻ 3-4 eggs per week	◻ > 4 eggs per week	◻ 1-2 eggs per month	◻ Never

15) How often do you eat legumes (beans, lentils, chickpeas, peas...)?

◻ 2 daily (lunch and dinner)	◻ 1 daily	◻ 3- 4 per week	◻ 1-2 per week	◻ Never

16) Which of these products do you use? (more than one choice is possible)

◻ Soy sauce	◻ Miso	◻ Sesame seed	◻ Tahin	◻ Gomasio	◻ Algae
◻ Linseed/sunflower seed/pumpkin seed	◻ Bran/whole bran	◻ Brewer's yeast (supplementation)	◻ Wheat germ	◻ Sprouts	

17) How often do you eat Seitan/Tofu/Tempeh?

◻ 2 daily (lunch and dinner)	◻ 1 daily	◻ 3- 4 per week	◻ 1-2 per week	◻ Never

18) How often do you eat vegetables?

◻ 2 daily (lunch and dinner)	◻ 1 daily	◻ 3- 4 per week	◻ 1-2 per week	◻ Never

19) How often do you eat fruit?

◻ 1 fruit daily	◻ 2 fruit daily	◻ 3-4-fruit daily	◻ 3-4 fruit per week	◻ 3-4 fruit per month	◻ Never

a) When do you eat fruit? (more than one choice is possible)

◻ Snack	◻ Post meal	◻ Other ________________________

20) How often do you eat dried fruit (almond, nuts, hazelnuts, pine nuts, pistachios, ...)?

◻ 2 daily	◻ 1 daily		◻ 1-2 per week	◻ Never

(lunch and dinner)		□ 3- 4 per week		

21) Do you eat sweets (candy, chewingum, chocolate, cakes, ice cream, croissant, snack etc.)?

□ Daily	□ 3-4- per week	□ 1-2 per week	□ 2-3- per month	□ Never

a) In which moment of the day do you eat sweets? (more than one choice is possible)

□ Breakfast	□ Lunch	□ Dinner	□ Snack	□ Fuori pasto

22) How often do you eat yogurt?

□ 1 daily	□ 2 daily	□ 2-4 per week	□ 2-3 per month	□ Never

a) Which type? (more than one choice is possible)

□ Natural yogurt	□ Fruit yogurt	□ Soy/Rice yogurt	□ Other ___________________________

23) Do you drink milk? □ YES □ NO

a) If YES, which type of milk do you drink? (more than one choices possible)

□ Full cream milk	□ Semi-skimmed milk	□ Skimmed milk	□ HD milk	□ Soy milk	□ Rice/oat/almond milk	□ Other ___________

b) How often do you drink milk?

□ Daily	□ 3-4 per week	□ 1-2per week	□ 2-3 per month	□ Never

24) Do you drink still water? □ YES □ NO Do you drink sparkling water? □ YES □ NO

a) How much water do you drink daily?

□ < ½ l	□ ½ l - 1 l	□ 1 l - 1 ½ l	□ > 1 ½ l

b) When you drink water? (more than one choice is possible)

□ During all day	□ During all day and at meals	□ only during the meals	□ Before trainig/racing	□ During training/racing	□ After training /racing
□Other ____					

25) How often do you drink sodas (cola, orange soda, soda, lemon soda, ice tea, … bottle or cans)?

□ Daily	□ 3-4 per week	□ 1-2 per week	□ 2-3 per month	□ Never

26) Beverages

a) Do you drink coffee? □ YES □ NO
- If YES, how many cups do you drink?

□ 1 cup	□ 2 cups	□ 3 cups	□ 4 cups	□ > 4 cups

b) Do you drink ginseng or barley coffee? □ YES □ NO

- If YES, how many cups do you drink?

□ 1 cup	□ 2 cups	□ 3 cups	□ 4 cups	□ > 4 cups

c) Do you drink tea? □ YES □ NO

- If YES, how many cups do you drink?

□ 1 cup	□ 2 cups	□ 3 cups	□ 4 cups	□ > 4 cups

d) Do you drink tea infusion or herbal tea? □ YES □ NO

- If YES, how many cups?

□ 1 cup	□ 2 cups	□ 3 cups	□ 4 cups	□ > 4 cups

e) Do you drink juices? □ YES □ NO
- If YES, how often do you drink juices?

□ Daily	□ 2-3 per week	□ 2-3 per month

f) Do you drink orange juice? □ YES □ NO

- If YES, How often do you drink orange juice?

□ Daily	□ 2-3 per week	□ 2-3 per month

27) Do you drink alcoholic beverages? □ YES □ NO
a) If YES, which type of alcoholic beverages (more than one choice is possible)?

□ Wine	□ Beer	□ Spirits

- How often do you drink WINE?

□ Daily	□ Weekend	□ 2-3 per week	□ 2-3 per month

- How often do you drink BEER?

□ Daily	□ Weekend	□ 2-3 per week	□ 2-3 per month

- How often do you drink SPIRITS?

□ Daily	□ Weekend	□ 2-3 per week	□ 2-3 per month

28) Which of these dressing do you use more? (more than one choice is possible)

□ Olio extra-vergine di oliva	□ Olio di oliva	□ Butter	□ Other ____________________

29) Do you often add salt? □ YES □ NO □ SOMETIMES
30) Which type of salt do you use? (more than one choice is possible)

□ Sea salt	□ Iodized salt	□ Unrefined sea salt	□ Other ____________________

31) How do you sweeten beverages and food (more than one choice is possible)

□Sugar	□Brown sugar	□ Honey	□Sweeteners (saccharin, aspartame, …)	□ Fructose	□ Other ____________________

a) How many spoons? (1 teaspoon= ½ spoon) daily?

□ 1 spoon	□ 2 spoons	□ 3 spoons	□ >3 spoons

32) Do you smoke? □ YES □ NO

a) If YES, how many cigarettes do you smoke daily? ____

Figure 1. Fruit Consumption of total sample, males and females at T1 and T3.

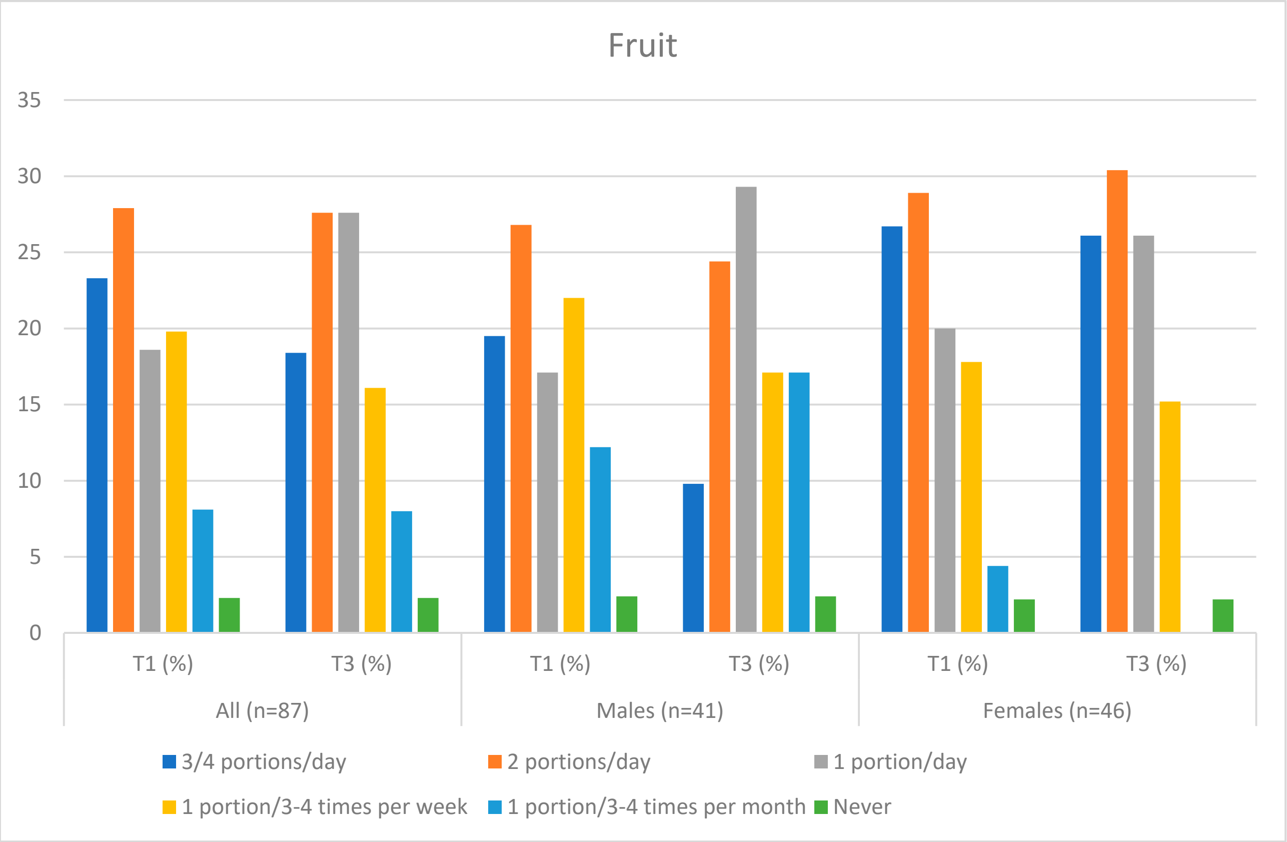

Figure 2. Vegetables Consumption of total sample, males and females at T1 and T3.

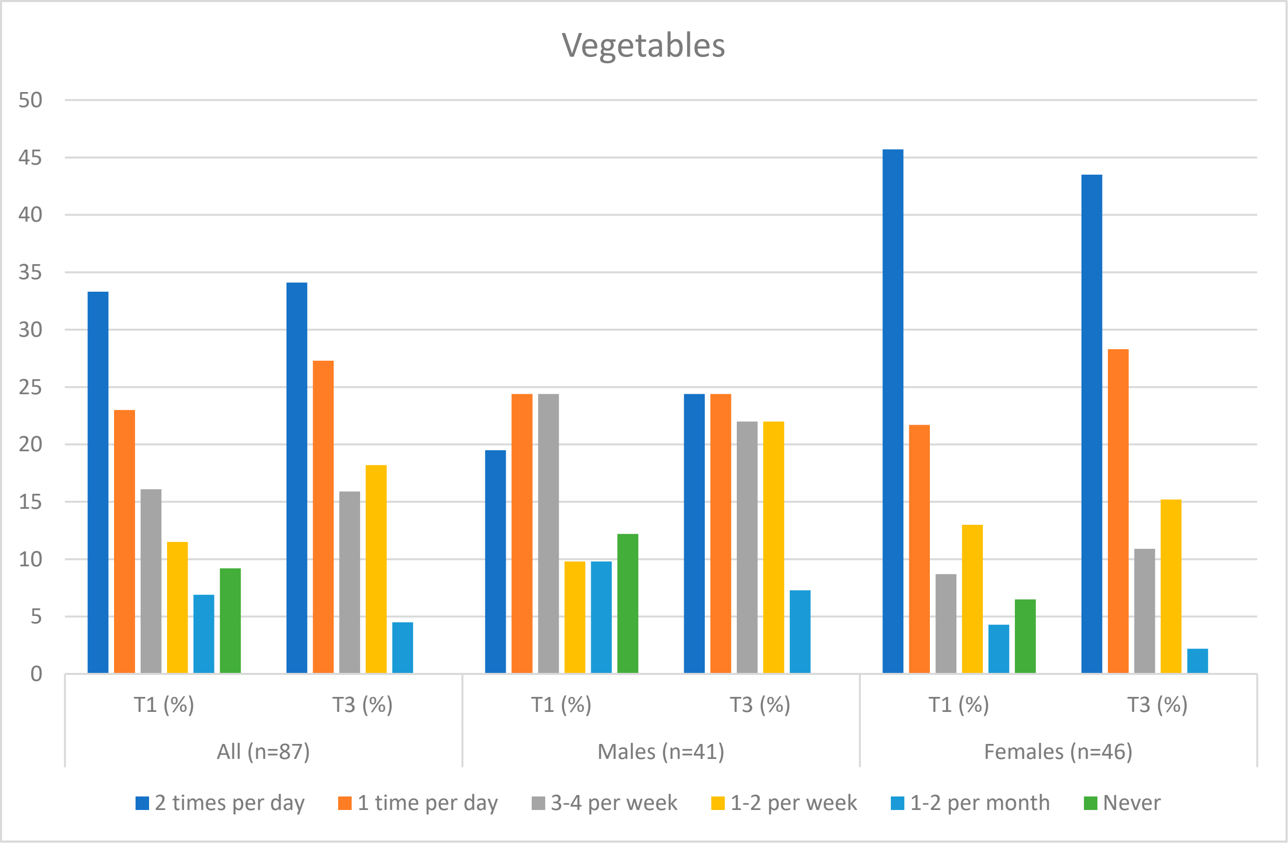

Figure 3. Legumes Consumption of total sample, males and females at T1 and T3.

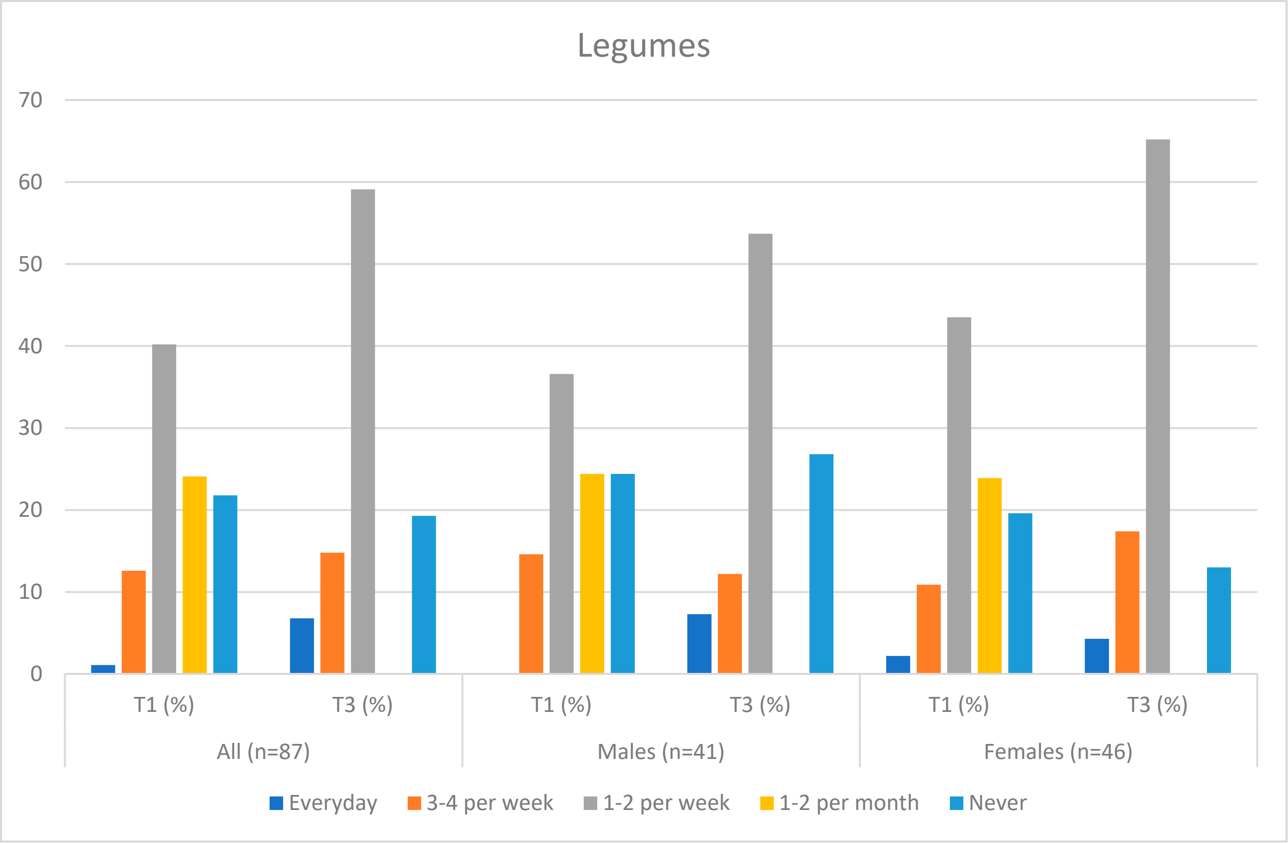

Figure 4. Fish Consumption of total sample, males and females at T1 and T3.

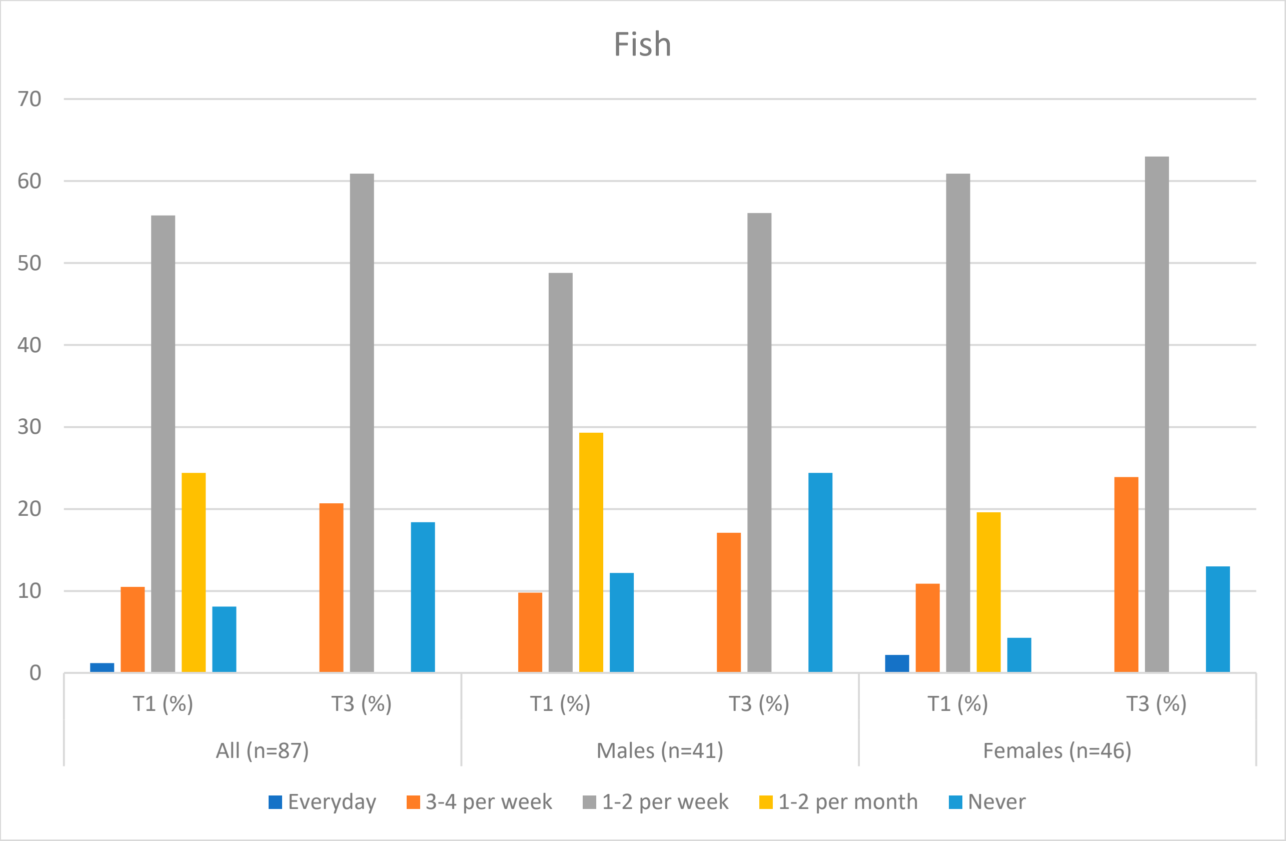

Figure 5. Meat Consumption of total sample, males and females at T1 and T3.

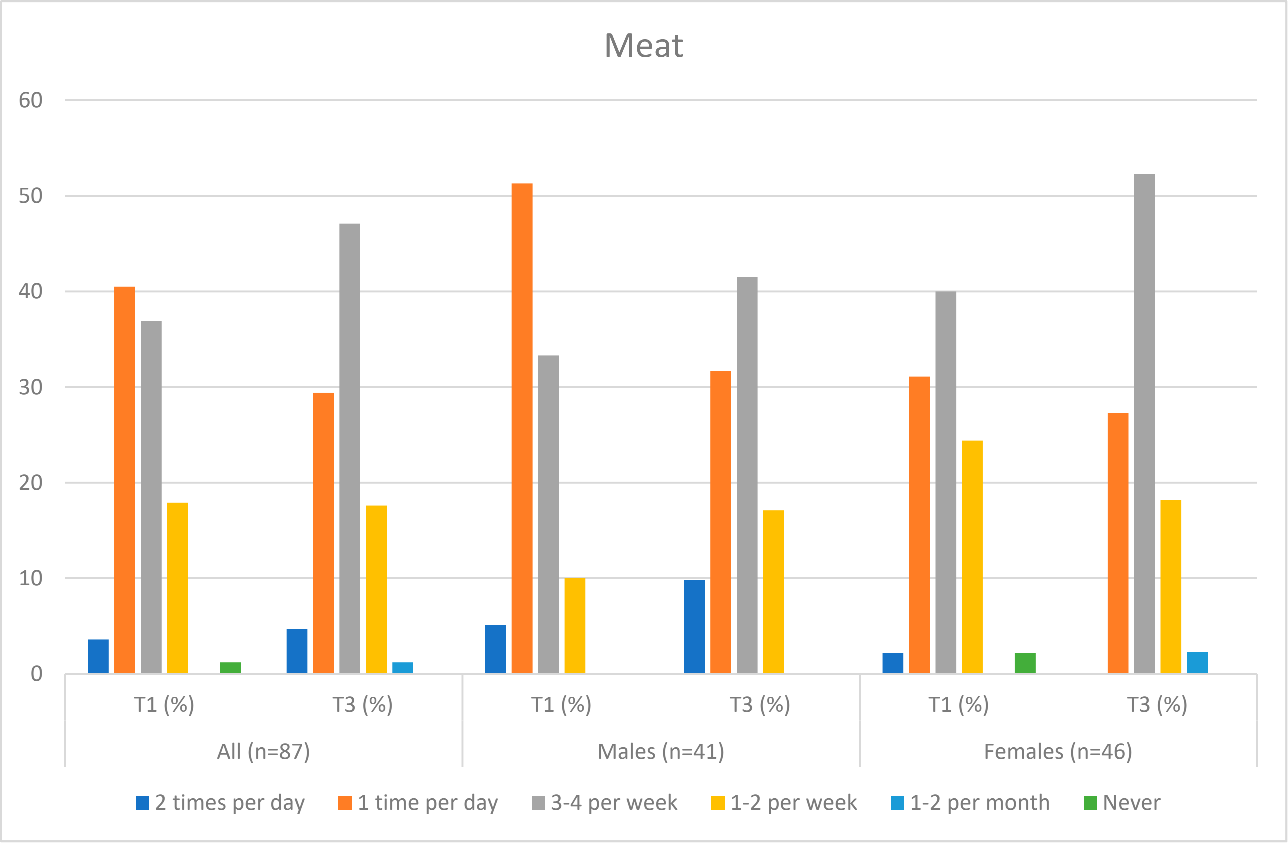

Figure 6. Nuts Consumption of total sample, males and females at T1 and T3.

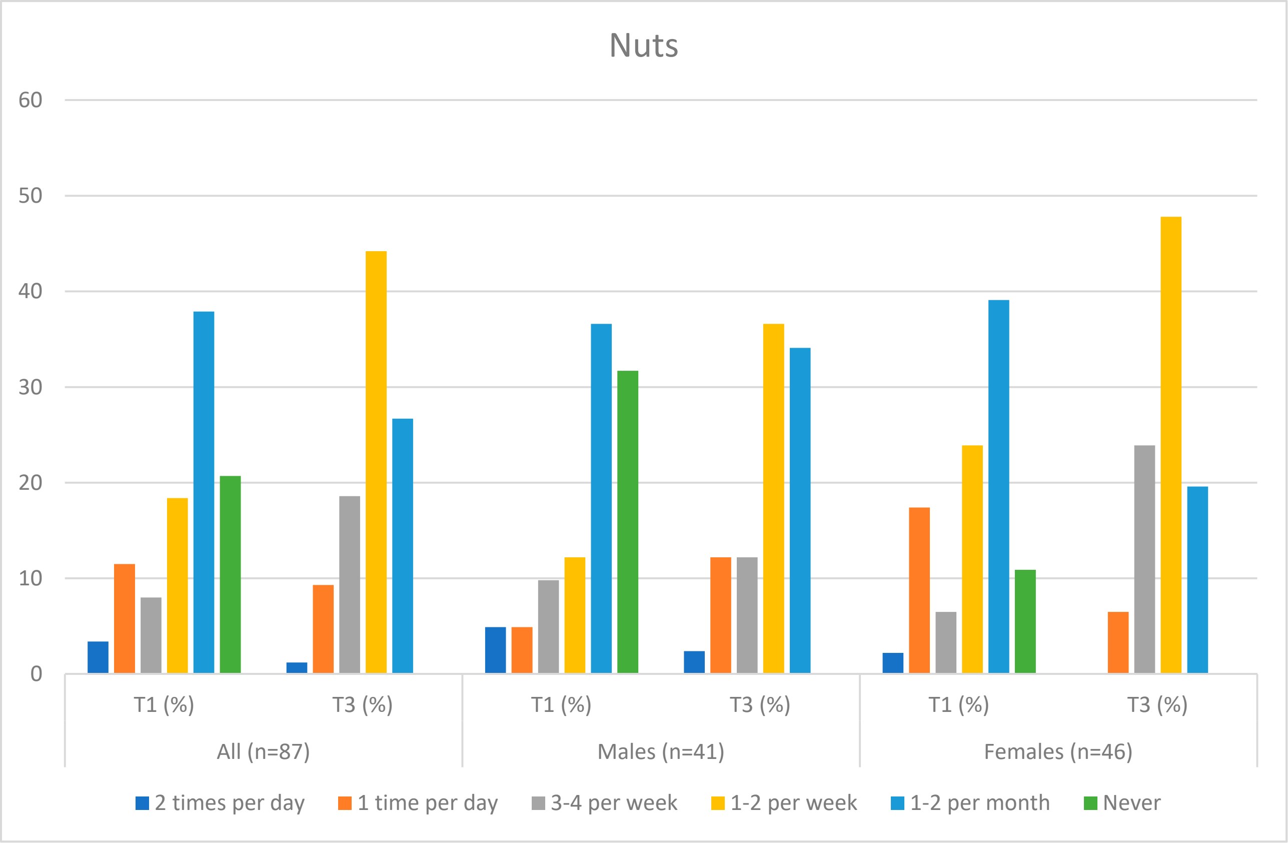

Supplement: Supplementary file 1 [file ijerph-18-07313-s001.zip › ijerph-1252214-supplementary.pdf]
